# *Drosophila* require both green and UV wavelengths for sun orientation but lack a time-compensated sun compass

**DOI:** 10.1242/jeb.246817

**Published:** 2024-10-14

**Authors:** Haneal Pae, Jingzhu Liao, Nicole Yuen, Ysabel Milton Giraldo

**Affiliations:** ^1^ Graduate Neuroscience Program, University of California, Riverside, Riverside, CA 92521, USA; ^2^ Department of Entomology, University of California, Riverside, Riverside, CA 92521, USA

**Keywords:** Sun compass, Insect, Flight, Celestial cue, Dispersal

## Abstract

Celestial orientation and navigation are performed by many organisms in contexts as diverse as migration, nest finding and straight-line orientation. The vinegar fly, *Drosophila melanogaster*, performs menotaxis in response to celestial cues during tethered flight and can disperse more than 10 km under field conditions. However, we still do not understand how spectral components of celestial cues and pauses in flight impact heading direction in flies. To assess individual heading, we began by testing flies in a rotating tether arena using a single green LED as a stimulus. We found that flies robustly perform menotaxis and fly straight for at least 20 min. Flies maintain their preferred heading directions after experiencing a period of darkness or stopping flight, even up to 2 h, but reset their heading when the LED changes position, suggesting that flies do not treat this stimulus as the sun. Next, we assessed the flies' responses to a UV spot alone or a paired UV–green stimulus – two dots situated 180 deg apart to simulate the solar and antisolar hemispheres. We found that flies respond to UV much as they do to green light; however, when the stimuli are paired, flies adjust for sudden 90 deg movements, performing sun orientation. Lastly, we found no evidence of a time-compensated sun compass when we moved the paired stimuli at 15 deg h^−1^ for 6 h. This study demonstrates that wavelength influences how flies respond to visual cues during flight, shaping the interpretation of visual information to execute an appropriate behavioral response.

## Teaser

**Summary:** In a flight arena, *Drosophila melanogaster* tracks sun stimulus movement in the presence of UV and green wavelengths but lacks a time-compensated sun compass.

## INTRODUCTION

Many animals – from bacteria to elephants – perform spatial orientation to find food, mates and hospitable environments. While the simplest changes in movement are undirected (kinesis), such as changes in speed or activity in response to changes in stimulus intensity (Ewer and Bursell, 1950), many locomotion strategies are directed. This directed movement can take many forms, from simple taxis to long distance migrations (Grob et al., 2021). Non-compass orientation occurs when animals use external or idiothetic cues that provide local rather than global directional information (Grob et al., 2021), such as the diel vertical migrations of zooplankton (Cohen and Forward, 2016), pheromone-mediated mate localization in silk moths (Takasaki et al., 2012) or straight-line orientation of dung beetles using celestial cues (Dacke et al., 2003, 2013, 2014; Foster et al., 2018). Indeed, celestial cues – the position of the sun or moon, polarized light, stars and the Milky Way – are some of the most ubiquitously used external cues by taxa during orientation (e.g. Dacke et al., 2013; Evangelista et al., 2014; Franzke et al., 2020; Mauck et al., 2008; von Frisch and Lindauer, 1956).

Monarch butterflies use sun position among other cues to direct their seasonal migrations (Merlin et al., 2009; Shlizerman et al., 2016), and honeybees are well known to use solar position to navigate to and from food sites (von Frisch and Lindauer, 1956). To extract real-world compass information from sun position, animals must possess an internal time-compensated sun compass to correct for the sun's movement across the sky (Cheeseman et al., 2012; Merlin et al., 2009; Shlizerman et al., 2016; von Frisch and Lindauer, 1956). Sun position is also used in shorter-distance navigational tasks, such as by desert ants of the genus *Cataglyphis* during their solitary foraging bouts. These ants can use the sun to provide compass cues, although they rely more heavily on the pattern of skylight polarization (Wehner and Müller, 2006). Some celestial cues can be used in non-compass orientation for organisms that lack a time-compensated sun compass (Grob et al., 2021). Instead they use celestial objects – such as the sun, moon or Milky Way – to perform menotaxis, adopting arbitrary headings with respect to an object (Heisenberg and Wolf, 1984). For example, dung beetles use the sun to perform menotaxis as they move their dung ball away from competitors at the dung pile (Byrne et al., 2003; Dacke et al., 2014). Similarly, tethered *Drosophila* perform menotaxis with respect to a green LED (Giraldo et al., 2018) or polarized light (Warren et al., 2018).

Beyond solar position, the sky contains additional visual information in the form of chromatic gradients and polarization pattern. Indeed, taxa vary in their cue preferences, such as diurnal dung beetles that prioritize celestial bodies for orientation, and nocturnal species that prefer polarized light (el Jundi et al., 2015b). Dung beetles orient to a green spot similarly to the sun (el Jundi et al., 2015b) and can use a variety of stimuli for orientation – green, ultraviolet (UV) or polarized light (el Jundi et al., 2016). Multiple studies across taxa demonstrate the utility of using simplified stimuli to assess responses to celestial cues (Beetz et al., 2022; Edrich et al., 1979; el Jundi et al., 2015a,b, 2016; Franzke et al., 2022).

Although *Drosophila* are not known to be migratory animals, field studies have shown that they can disperse long distances over inhospitable terrain (Coyne et al., 1982; Leitch et al., 2021). A population genetics and field study of *Drosophila pseudoobscura* indicated that the flies can travel over 10 km in 24 h, explaining the lack of genetic diversity among seemingly distinct populations (Jones et al., 1981). In an extension of this work, researchers fluorescently marked and released tens of thousands of *Drosophila melanogaster* and *Drosophila simulans* en masse in Death Valley, USA, on a March evening (Coyne et al., 1982). The following morning, flies were recovered from baited traps. Estimates of fly energetics indicate that *D. melanogaster* can fly for approximately 2 h before running out of energy (Götz, 1987) and the fact that flies did not have opportunity to refuel in the desert between the release site and the traps led to the conclusion that flies in the Coyne et al. (1982) study most likely flew straight, completing the journey over the course of only a few hours (Dickinson, 2014). These flies could have achieved this straight-line orientation by performing menotaxis using the sun or polarized light (Dickinson, 2014). More recently, Leitch and colleagues (2021) performed multiple desert releases with baited traps and cameras. Based on their shorter distance experiments and modeling, they concluded that *D. melanogaster* were probably capable of dispersing 12 km in still air and farther with wind assistance, likely using celestial cues to perform menotaxis (Leitch et al., 2021). Together, these studies suggest that *D. melanogaster* could use sun orientation to travel over 10 km. However, we have yet to understand their individual-level behavior in depth, as tracking individuals dispersing in the field is constrained by their small body size, in which the smallest radio telemetry tags (5×5 mm) exceed the body length of *Drosophila* (Kumari and Hasan, 2020)*.* Furthermore, we have a limited understanding of how different visual cues shape a decision to change direction or go straight.

To understand sun orientation in *D. melanogaster* in which flies can move more naturalistically than under rigid tether conditions, we tested flies in a flight arena in which the fly can rotate freely on its yaw axis (Bender and Dickinson, 2006; Weir and Dickinson, 2012). By varying flight duration, angular movement of the stimulus and cue wavelength, we sought to uncover the fidelity of heading maintenance in *D. melanogaster*. We hypothesized that sun orientation is robust to perturbation and is maintained during long periods of flight. We expected flies to adjust for a change in stimulus position, providing evidence for a sun compass. Overall, our findings show that tethered *Drosophila* robustly perform menotaxis in response to a green or UV spot, but do not track the movement of these stimuli. In contrast, when we presented paired solar and anti-solar stimuli, reflecting a more realistic celestial cue, flies tracked the stimuli movements, thus performing sun orientation. These results indicate that flies are sensitive to stimulus wavelength. Furthermore, when we examined whether flies possess a time-compensated sun compass in response to either the single green or paired green–UV stimuli, we found no evidence of time compensation, consistent with a hypothesis that flies do not require this behavior in the wild. Our work provides insight into how visual inputs influence heading maintenance during simple menotaxis and sun orientation in *Drosophila*, which will pave the way for future investigation into the neural basis of heading preference and the processing of complex visual cues in non-migratory insects.

## MATERIALS AND METHODS

### Animals

Experiments were conducted using 2–4 day old female wild-type *Drosophila melanogaster*. All experiments were conducted on a Heisenberg Canton-S (HCS) line obtained from the Dickinson lab (California Institute of Technology, USA). Flies were reared on standard cornmeal diet in an incubator maintained at 25*°*C on a 12 h:12 h light:dark cycle. In all of our tests, each fly was tested only once, and any fly that stopped more than 2 times per flight period was discarded.

### Fly tethering

We tethered flies to a 0.1 mm diameter magnetic pin (Living Systems Instrumentation, PIN-0.1MM) by UV-cured glue (Bondic, #SK8024) under cold anesthesia. Before behavioral testing, flies recovered from anesthesia for at least 10 min. To prevent flies from exhausting their energy reserves, prior to flight experiments, we provided each fly with a small piece of paper to induce a landing reflex until the experiment began. All flies were tested within 1 h of tethering.

### Flight arena

Orientation during flight was tested using a flight simulator arena where they rotate freely on the vertical axis in yaw (hereafter, rotating tether arena), modeled on previously published designs (Bender and Dickinson, 2006; Weir and Dickinson, 2012). We selected a rotating tether arena as this provides more naturalistic flight and, importantly, it allows us to control the position and rate of movement of the stimulus to test for the ability to track a stimulus during sun orientation and the presence of a time-compensated sun compass. Tethered flies were placed individually in the arena and held between two magnets (Grainger #10E804; Magcraft NSN0750). The pin made contact with a ruby jewel bearing (Microlap Technologies, #MT4019, gift from Michael Dickinson, California Institute of Technology, USA) creating a low friction surface that allowed free rotation about the fly's vertical axis. The fly was backlit with a custom-built IR LED PCB (peak λ=940 nm, courtesy of Will Dickson, https://github.com/willdickson/magneto_ir_led_ring). We tracked the fly's heading in real time using a camera (Blackfly S USB3 monochrome camera, BFS-U3-16S2M-CS) with macro lens (Computar MLM3X-MP), and we extracted body orientation using custom Python 2.7.18 scripts, courtesy of Will Dickson (https://github.com/willdickson/find_fly_angle.git). To simulate the sun, we initially selected a single LED from an RGB LED strip (Adafruit #1506, λ=510 nm) set to green, the dominant wavelength of sunlight. A single green LED is most commonly used to simulate the sun in laboratory experiments (el Jundi et al., 2015a,b, 2016; Beetz et al., 2022).

Next, we set out to test whether flies exhibit the same behavior to a UV stimulus, another chromatic component of sunlight that is visible to many insects, including *Drosophila* (Weir and Dickinson, 2012). We added a UV LED strip (Adafruit #5722, λ=395 nm) above the RGB LED strip. Previous insect navigation studies have used a similar setup to simulate the sun, where a single LED from an RGB LED strip was used (Beetz et al., 2022; el Jundi et al., 2016) as insects are shown to treat artificial light similarly to the sun (Edrich et al., 1979; el Jundi et al., 2015b). Both LED strips entered the arena through a small hole, which was fully sealed with electrical tape to prevent light from entering the arena. We covered all interior surfaces of the arena except for our LED strips with black flocking to minimize reflections in the arena (Edmund Optics, #54-585). To determine the contrast of our green LED, we measured the normalized brightness for the brightest and darkest areas in our arena at three angular positions (15, 90, 180 deg) from the LED (Thorlabs light meter and sensor, S130C, PM100A). In all cases, we measured a Michelson contrast of 1.0 indicating that our stimulus provided a high contrast point source of light. For the UV LED stimulus, we used the same brightness intensity as the green LED. During each flight trial, the LED was set at random to one of four positions that were 90 deg apart using an Arduino Nano microcontroller (#A000005) and custom Python scripts (https://github.com/willdickson/basic_led_strip, https://github.com/willdickson/basic_led_strip_ros, https://github.com/GiraldoLab/Magnotether-Arena/blob/main/shuffle_sun_node.py).

### Long-term flight trials

To examine orientation behavior in a rotating tether arena, we conducted experiments which began with a period of darkness (30 s) and a 20 min flight period. To see whether flies adjusted their heading when the LED position changed, we also conducted flight trials with a period of darkness (30 s) and a 20 min flight period during which the LED's position changed every 5 min by 90 or 180 deg. The 30 s dark period is characterized by rapid body rotations which were used to confirm proper tethering and the absence of extraneous visual stimuli during the experiment, consistent with longer duration trials in the dark (Weir and Dickinson, 2012). Any fly that did not rotate freely during the 30 s dark period was discarded as a tethering error. The majority of flies were expected to fly for the full duration of the trial (Weir and Dickinson, 2012); however, if the fly stopped mid-trial, it was given an air puff to continue its flight. Flies stopping more than 2 times per flight period were excluded from the final dataset.

### Varied stimuli flight trials

To assess how visual and motor stimuli differentially affect heading direction, we conducted experiments in which we varied the LED position and the visual environment (dark or with the presence of an illuminated LED) and motor state (flight or rest) of the fly between two bouts of flight. All experiments began with a period of darkness (30 s), a flight period (5 min), a varied stimulus period (5 min), and a second flight period in which the LED position was either the same or different from the LED position in the first flight period (5 min). During the varied stimulus period, the fly continued to fly or was provided with a period of rest (interrupted flight). We induced a landing reflex with a small piece of Kimwipe. For each of these two motor state conditions, we provided two different visual stimuli: darkness or an illuminated LED. For experiments in which we did not change the LED position, the LED was kept in the same position during the intertrial period. In experiments in which we changed the stimulus position, the LED was set to a new position 90 or 180 deg from the original position during the intertrial period, resulting in three LED positions during the course of the experiment. We used the data collected during long-term flight trials to examine heading changes when the LED was maintained in the same position and the fly flew continuously. In this case, the first headings were calculated for minutes 0–5 and 10–15 of the 20 min trial. As in the long-term flight trials, if the fly stopped mid-trial, it was given an air puff to continue its flight. Flies stopping more than 2 times per flight period were excluded from the final dataset.

### Time gap experiments

To explore how long flies can maintain their heading in the rotating tether arena, we tested whether they changed heading direction with varying periods of rest between two bouts of flight. All experiments began with a period of darkness (30 s), a first flight period (5 min), a 1, 2, 6 or 8 h period of rest, and a second flight trial, with the LED in the same position as the first flight period (5 min). LED positions were selected at random from the four starting positions as in long-term flight trials. During the rest period, flies were removed from the arena, given a piece of Kimwipe and placed in an empty fly vial, covered with flocking paper to ensure darkness. The top of the fly vial was dipped in water and a Petri dish with water was kept nearby to prevent desiccation. Each fly was tested only once, and data from any fly that stopped more than 2 times per flight period were discarded.

### Small and gradual stimulus shift trials

To determine whether flies would maintain the same heading relative to our green LED when it was moved a smaller angle, we presented flies with 30 s of darkness, 5 min of flight, a second flight period when the LED shifted 15 deg clockwise or counterclockwise (5 min), and a third flight period when the LED shifted another 15 deg in the same direction as the previous flight trial (5 min). For these small stimulus shift flight trials, the stimulus starting position was selected from any LED position of the LED strip that allowed the full 30 deg of LED positions, without encountering the LED strip entrance, where the distance and position of the LEDs differed slightly from the rest of the arena to account for the wiring of the LED strip.

Next, we tested whether flies could track the LED position when we moved the stimulus gradually. We presented flies with 30 s of darkness, and then 12 consecutive LED presentations of 100 s each, shifting the LED either clockwise or counterclockwise by ∼5 deg each time, for a total of 60 deg movement for the duration of the experiment. As above, the starting position was selected from any LED position which allowed 60 deg of positions that did not cross the LED strip entrance to the arena.

### Testing for a time-compensated sun compass in response to a green LED

To examine whether flies possessed a time-compensated sun compass, we tested whether flies would adjust their heading to the rate of 15 deg h^−1^, matching the rate of the sun's actual movement over the course of 6 h. Each flight trial began with a brief (30 s) period of darkness and a 5 min flight period with a green LED as a sun stimulus. Each fly was subsequently tested 3 more times: 1, 2 and 6 h after the first flight trial. Between each flight trial, we gave flies a piece of Kimwipe to stop flight and placed them individually in an empty fly vial, as in the time gap experiments described above. Each fly was randomly assigned to one of two starting positions and was presented with either clockwise or counterclockwise movement for the duration of the trial. Flies were discarded if they stopped more than twice during a flight trial as in previous experiments.

### Testing for cue preference with paired green and UV cues

To simulate the spectral properties of the solar and anti-solar hemisphere of the sky and to assess flies' responses to these paired cues, we added a UV LED 180 deg apart from the green LED. We presented both LED cues 180 deg apart for a 5 min flight period and then turned off one of LEDs for a second 5 min flight period without a break in between, similar to a study with dung beetles by el Jundi et al. (2015a). While the presence of two illuminated LEDs increased the total brightness in the arena, heading and vector strength were similar to single LED presentations. Therefore, in our paired stimuli trials, we maintained the same individual LED brightness as in single LED experiments. Flies were discarded if they stopped more than twice during a flight trial as above.

### UV LED stimulus movement trials

As flies preferred to orient to the UV LED rather than the green LED when initially presented with both, we tested whether flies would better maintain their heading relative to a UV LED when the UV LED changed position by 90 or 180 deg. We presented flies with a 30 s period of darkness followed by a 5 min flight period with a UV LED followed by a second 5 min flight period when we rotated the position of the illuminated UV LED by 90 or 180 deg clockwise or counterclockwise. The clockwise/counterclockwise direction was determined at random. The starting UV LED was selected at random from four pre-selected positions set 90 deg apart. Data from flies that stopped more than 2 times were discarded as above.

### Paired green and UV stimuli movement trials

To determine whether flies required both green and UV spectral cues to maintain their heading during a 90 deg LED position change, we used both green and UV LEDs which were set 180 deg apart. In the flight arena, flies were presented with a 30 s period of darkness, a 5 min flight period with both LEDs set 180 deg apart, followed by a second 5 min flight period when the green and UV LEDs were both moved 90 deg clockwise or counterclockwise with the direction of movement selected at random. The green and UV LED pairing were selected at random from four pre-selected positions which were 90 deg apart. Data from flies that stopped more than 2 times during the trials were discarded.

### Testing for a time-compensated sun compass in response to paired green and UV LEDs

To examine whether the lack of a time-compensated sun compass that we observed in response to a green LED was due to the limited spectral information, we used paired LEDs as described above. Both LEDs were moved at the rate of the sun's actual movement, 15 deg h^−1^, as we had tested for a single green LED. Each flight trial began with a brief (30 s) dark period followed by a 5 min flight period with paired UV–green stimuli. Each fly was subsequently tested 3 more times: 1, 2 and 6 h after the first flight trial. Flies were given a rest period in a dark chamber as described in the time-compensation experiment with a single green LED. Two sets of LED positions were used, one for the clockwise and the other for the counterclockwise movement of the stimuli. To further investigate whether flies possessed a time-compensated sun compass, we used datasets from our trials with the green LED alone and data collected here, and separated the data depending on the direction of LED movement – clockwise or counterclockwise. If flies compensated for the LED movement, the total heading change over the course of 6 h would be clustered around −90 deg for the clockwise group and 90 deg for the counterclockwise group. Data were discarded if the fly stopped more than twice during a flight trial, as in previous experiments.

### Data and statistical analysis

To examine what factors lead to changes in heading during flight, we used both descriptive statistics and statistical tests for circular data. For descriptive statistics, we processed and analyzed data using custom Python 2.7 analysis code (Giraldo et al., 2018) and Circstats (https://github.com/jhamrick/python-snippets/blob/master/snippets/circstats.py), a Python package used to analyze circular data. For each flight trial, the headings of the fly were collected in a csv file at the frame rate of the camera (25 frames s^−1^). We used a custom data analysis code to calculate the circular mean angle and the circular variance for each trial (https://github.com/GiraldoLab/JEB_2024). To assess how persistently the fly continued with its preferred heading during a flight period, we calculated the flies' vector strength, the vector sum of all of the fly's instantaneous headings. A vector strength of 1 indicates that the fly did not change its heading at all, whereas a vector strength closer to 0 indicates that the fly frequently changed its heading direction (Berens, 2009; Zarr, 1999). Population circular variance depicts the spread of circular data with a value between 0 and 1, a unitless metric. Values closer to 1 indicate widely distributed data (Berens, 2009). Flies with a vector strength smaller than 0.2 per flight period were excluded because the calculated mean heading for these individuals was not meaningful, as they did not select a heading during the flight trials. However, this exclusion occurred in only 4.3% of flight trials, indicating that the overwhelming majority of flies selected a heading direction. This cutoff slightly changed sample sizes in subpanels in each figure. To quantify the degree of change in heading for each flight trial, we found the differences between absolute mean headings for each flight period (heading difference). When comparing whether flies in our small stimulus shift and gradual stimulus shift experiments tracked the LED position or maintained their heading with respect to the arena, we calculated both the mean heading angles relative to the LED and relative to the arena (real-world heading) for each flight period. The custom data analysis code (https://github.com/GiraldoLab/JEB_2024) also characterized the population mean heading, population variance and a confidence interval (CI) of 95%. If the variance of the data was larger than 0.8, indicating a wide spread of data, the CI could not be calculated and was not plotted in our figures. As the CI values cannot be reported for some datasets, we reported the circular variance for all datasets.

Statistical tests for circular data were used to compare heading differences between different experimental groups using the CircStat package in MATLAB R2022b. In order to test for circular uniformity, we used a Rayleigh test (Berens, 2009; Zarr, 1999). To test whether the different experimental conditions caused a change in mean heading during long-term flight trials, we used a Watson–Williams test which tests for a significant difference in mean direction between two groups (Berens, 2009).

For our varied stimuli trials, we used a general linear model adapted for circular statistics (Cremers and Klugkist, 2018) to explore how the change in LED position, visual stimuli (dark and an illuminated LED) and motor state (rest and flight) affected heading maintenance. To examine whether the magnitude of the LED movement influenced the change in heading direction, we ran a GLM on only the datasets in which the LED position moved, using magnitude of stimulus position (90 or 180 deg), dark or LED, and rest/flight as factors. We used R (R version 4.3.1) and the ‘circular’ package to test how our independent variables (LED position change, visual stimuli and motor state) affected the dependent variable (heading difference). To further confirm whether the maintenance of heading was likely to occur by chance alone, we used a reshuffling technique as in Giraldo et al. (2018). In brief, a shuffled list of absolute mean headings of the second flight period was paired with the list of absolute mean headings of the first flight period and the mean absolute heading difference was calculated. This process was repeated 10,000 times to produce a shuffled distribution of absolute mean heading differences. We calculated the proportion (*P*-value) of the reshuffled mean headings that were smaller than the observed mean heading difference of the collected dataset. This resampling approach was also used to test for a change in heading in our time gap experiments.

In our small stimulus shift and gradual stimulus shift experiments, we used a Watson's *U*^2^ test to test whether the distributions and means of absolute and relative heading changes differed statistically (Watson, 1962).

### Use of AI

ChatGPT from OpenAI was used to edit and optimize data analysis code to improve analysis efficiency and provide a more user-friendly experience (https://github.com/GiraldoLab/JEB_2024). We also used ChatGPT to assist with editing code that controlled the presentation of the LEDs by adding LED positions used in the gradual LED presentation experiments (https://github.com/GiraldoLab/Magnotether_Arena/blob/main/led_strip_magnotether/shuffle_sun_node). Subsequently, we used ChatGPT to generate user instructions to run the GLM analysis in R.

## RESULTS

### Flies fly straight with respect to a sun stimulus in a rotating tether arena

**Fig. 1.**
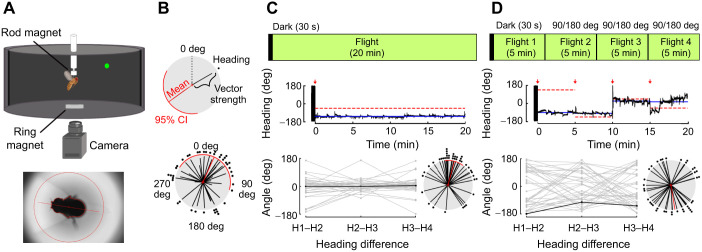
**Flies perform menotaxis to a green LED held in a constant position.** (A) Top: schematic diagram of the rotating tether arena used for flight trials. A tethered fly was held between rod and ring magnets, and the fly's heading was recorded from a camera below. Bottom: example tracking image of a fly. (B) Top: schematic diagram of the polar plot used to show flies' headings. Each black line and dot represents the fly's mean heading angle relative to the LED (set to 0 deg). Line length represents vector strength. Population mean heading angle and 95% confidence interval (CI) are indicated in red, when possible to calculate such a value. Bottom: headings of flies during a 20 min flight with one continuous LED (*n*=41, mean±95% CI, variance). (C) Experimental paradigm for long-term flight trials with a representative example of a fly's heading. Time is indicated on the *x*-axis and heading angle is shown on the *y*-axis. LED position is indicated by the dashed red line, LED onset by the red arrow, and the mean heading for the trial is indicated by the solid blue line. Heading differences calculated for each 5 min period are plotted as a linear plot (bottom left) with the last minus first 5 min heading difference plotted as a polar plot (bottom right, *n*=41, mean±95% CI, variance). (D) Experimental paradigm of 20 min flight where the LED position changed every 5 min (red arrows). Heading differences calculated for each 5 min period were plotted as a linear and polar plot as in C (*n*=38, mean±95% CI, variance).

To study sun orientation in a laboratory setting where we can continuously track individual heading during flight with a visual stimulus, we built a flight arena where flies can freely rotate in yaw, simulating more naturalistic flight (Fig. 1A; Bender and Dickinson, 2006; Weir and Dickinson, 2012). To characterize flies' responses to a stimulus often used to simulate the sun, we presented a single green LED at one of four preselected positions spaced 90 deg apart and recorded the flies' heading for 20 min (Fig. 1B; *n*=41, mean=24.3 deg, variance=0.78). We found that flies selected an arbitrary heading relative to the LED (Fig. 1C) and generally adopted straight paths (*n*=41, mean=15.1 deg, variance=0.46). To assess consistency in heading during a 20 min flight, we partitioned flight trials into 5 min intervals (H1–H4), calculated the change in heading for each 5 min (Fig. 1C), and found that individuals maintained their headings. To test whether flies maintained their heading in response to LED movements, we changed the LED's position every 5 min by either 90 or 180 deg from the randomly selected starting position, expecting the fly to track the stimulus to maintain its heading relative to the stimulus. However, we found that flies changed their headings with each LED position change (Fig. 1D; *n*=38, mean heading change=170.5 deg, variance=0.85). To statistically assess whether this change in heading differed significantly when we either kept the LED position constant or moved it by 90 or 180 deg, we calculated the heading difference between the first and last 5 min for both 20 min trials and found a significant difference in mean heading direction between the two groups (Watson–Williams *F*=20.46, *P*<0.01). We also tested whether there was a difference in mean heading for each 5 min period between the two 20 min trials, and saw a significant difference in mean heading direction, after performing a Benjamini–Hochberg false discovery rate correction (Watson–Williams H2–H1: adjusted *P*<0.001, H3–H2: adjusted *P*=0.017, H4–H3: adjusted *P*<0.001).

Because heading can persist in the dark and drift over time (Seelig and Jayaraman, 2015), we asked whether this change in heading was due to drift. We calculated drift over a 5 min flight by examining the difference in heading between the mean heading of the initial 0–5 min and the mean heading of the 10–15 min period, as in Warren et al. (2018). When the stimulus position was held constant, flies exhibited 6.9 deg drift. In contrast when the stimulus was moved by 90 or 180 deg, the mean heading difference was 115.7 deg. This suggests that the heading change over the same time period is likely because of the change in LED position. Additionally, we examined whether flies maintained their heading in the arena (real-world heading) in response to moving the green LED. We found that although flies tended to maintain their real-world heading regardless of the magnitude of LED movement, 90 or 180 deg, heading change increased with subsequent stimulus movements (Fig. S1; *n*=38, H2–H1: mean=2.8 deg, variance=0.44, H2–H1: mean=4.4 deg, variance=0.76, H4–H3: mean=149.6 deg, variance=0.88, H4–H1: mean=27.4 deg, variance=0.92).

### Flies maintain heading direction when flight is interrupted and LED position is unchanged

**Fig. 2.**
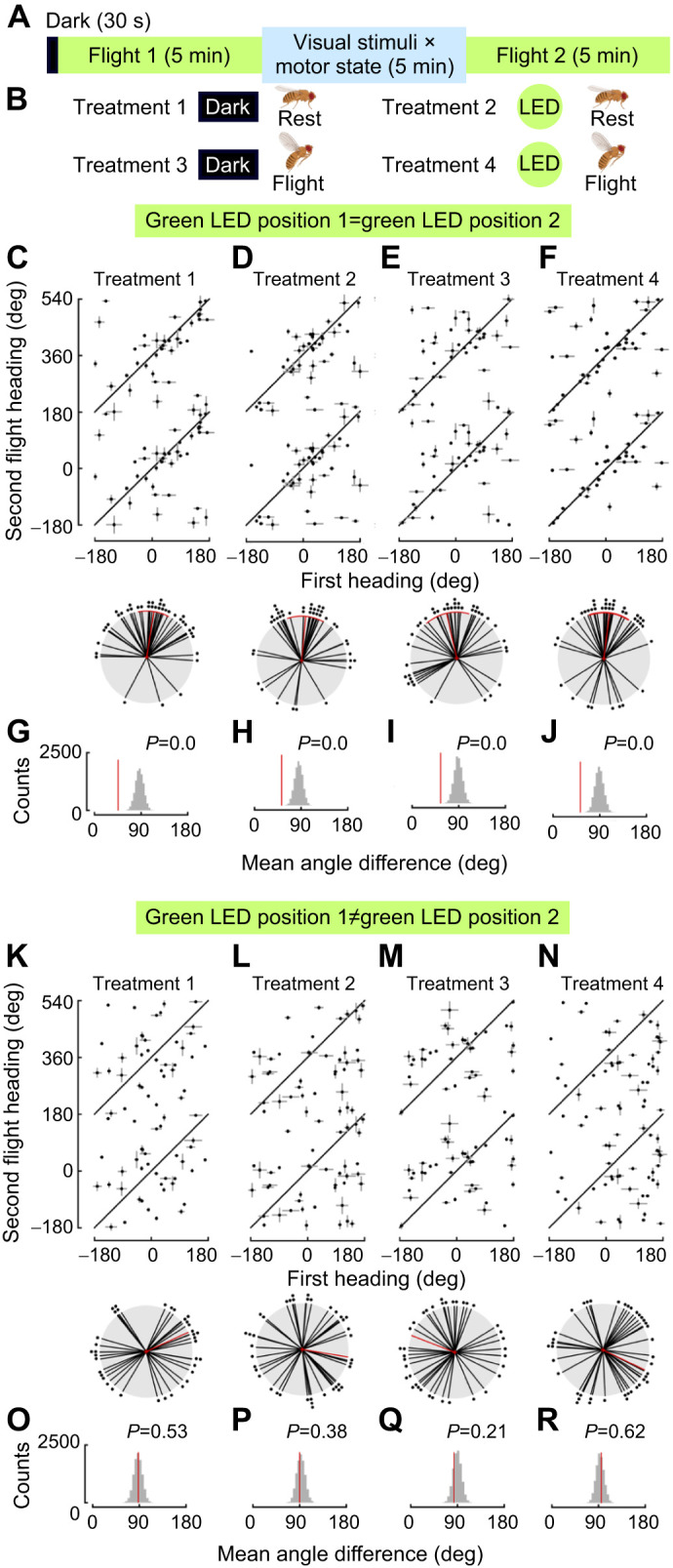
**Flies maintain heading direction during continuous and interrupted flight when the green LED position remains the same.** (A) Experimental paradigm for the varied stimuli flight trial. (B) Four different treatment groups were used for the intertrial period combining two different visual states and motor states: dark–rest, LED–rest, dark–flight and LED–flight. (C–F) Linear plots of heading during flight when the LED position does not change – the first heading is plotted on the *x*-axis and the second flight heading is plotted on the *y-*axis. Data are repeated on the vertical axis to account for their circular nature. The solid black diagonal line indicates where points would lie if all flies maintained the same heading direction. Error bars show the variance for each flight, scaled for visibility by multiplying with an arbitrary number, 36. Heading differences between the first and second flight are plotted as polar plots, using the same plotting conventions as in Fig. 1B (bottom, mean±95% CI, variance; dark–rest *n*=40, LED–rest *n*=41, dark–flight *n*=40, LED–flight *n*=39). (G–J) Reshuffled distribution of heading differences through 10,000 random pairings of first and second flights. The red line indicates the absolute mean heading difference of the data collected. The gray histogram indicates the distribution of reshuffled headings. The *P*-value indicates the proportion of absolute mean heading differences that are smaller than the observed data. (K–N) Heading differences for the varied stimuli flight trials when the LED position changed between Flight 1 and Flight 2 (mean±95% CI, variance; dark–rest *n*=37, LED–rest *n*=40, dark–flight *n*=37, LED–light *n*=41). (O–R) Resampling results of heading data shown in K–N.

To test whether a period of rest or darkness would also cause a change in heading, we designed a paradigm in which we presented flies with a green LED for 5 min (Flight 1) followed by a varied stimulus period, and a second green LED (Flight 2; Fig. 2A). The varied stimulus period consisted of two different visual states (dark or an illuminated LED) and two motor states (flight and rest; Fig. 2B). In addition, we varied whether flies experienced the LED in the same position or moved by 90 or 180 deg during the second LED presentation. This combination of parameters allowed us to test the role of LED movement as well as the flies' experience during the varied stimulus period. During both flights, flies adopted arbitrary headings and the majority of flies flew straight (all individuals had a vector strength greater than 0.2), as in our long-term experiments (Fig. 1). When the LED was maintained in the same position in the first and second LED presentation bouts, we found that flies did not change their heading (Fig. 2C–F, dark–rest: mean=8.7 deg, variance=0.39, *n*=40; LED–rest: mean=5.2 deg, variance=0.49, *n*=41; dark–flight: mean=−12.8 deg, variance=0.55, *n*=40; LED–flight: mean=5.2 deg, variance=0.5, *n*=39; example individual data; Fig. S2). To examine whether flies changed heading from the first to the second flight, we first adopted a reshuffling approach as in Giraldo et al. (2018). When the LED position was unchanged, in all cases the observed data had a smaller heading difference to the reshuffled data, suggesting that these patterns were unlikely to have occurred by chance (Fig. 2G–J, all *P*=0.0). In contrast, when the LED position was changed by 90 or 180 deg, the change in heading was not centered on zero (Fig. 2K–N, dark–rest: mean=65.5 deg, variance=0.91, *n*=37; LED–rest: mean=99.5 deg, variance=0.96, *n*=40; dark–flight: mean=−68.8 deg, variance=0.81, *n*=37; LED–flight: mean=115.7 deg, variance=0.85, *n*=41; example individual data; Fig. S3). When we examined the data from trials in which we moved the LED position, flies' change in heading was not significantly smaller than in reshuffled datasets (Fig. 2O–R, all *P*>0.2). To test whether other variables that we manipulated had an effect on heading maintenance in *Drosophila*, we performed a circular analog of a generalized linear model (GLM) (Cremers and Klugkist, 2018). The GLM confirmed that the only significant factor that influenced heading maintenance was whether or not we changed the position of the LED (GLM: LED position change: *P*=0.001, flight/rest: *P*=0.29, dark/LED: *P*=0.1). Indeed, to be sure that GLM variable order did not influence the significance of our results, we ran the GLM with all possible variable orders and found in all cases that the only significant variable was LED position (all *P*<0.001 for LED position, other variables *P*>0.1). These experiments demonstrated that flies robustly maintained their heading direction even when stopping flight or experiencing a period of darkness but changed heading with respect to the stimulus in response to large changes of our green LED.

Lastly, we wanted to understand whether the magnitude of stimulus movement affected the magnitude of the fly's heading change. We segregated the data in Fig. 2K–N by whether the stimulus moved 90 or 180 deg and found that the angle of the LED movement affected heading change with a marginal effect of whether the LED was illuminated between the two flight trials (Fig. S4; GLM: LED position change: *P*=0.004, flight/rest: *P*=0.304, dark/LED: *P*=0.05). Indeed, flies appeared to select a new heading when the LED was moved 90 deg (Fig. S4; dark–rest: mean=53.4 deg, variance=0.64, *n*=22; LED–rest: mean=7.9 deg, variance=0.89, *n*=27; dark–flight: mean=−64.4 deg, variance=0.64, *n*=29; LED–flight: mean=73.1 deg, variance=0.74, *n*=28). In contrast, flies tended to maintain their real-world headings when the LED position changed by 180 deg (Fig. S4; dark–rest: mean=−134.5, variance=0.68, *n*=15; LED–rest: mean=164.9 deg, variance=0.53, *n*=11; dark–flight: mean=123.6 deg, variance=0.53, *n*=8; LED–flight: mean=−164.8 deg, variance=0.61, *n*=13). We found no consistent pattern of heading change when flies were in the dark during the intertrial interval and given the relatively small sample size for 180 deg changes, we conclude that the presence or absence of light in the intertrial interval is not a critical factor during orientation.

### Flies maintain heading direction for hours

**Fig. 3.**
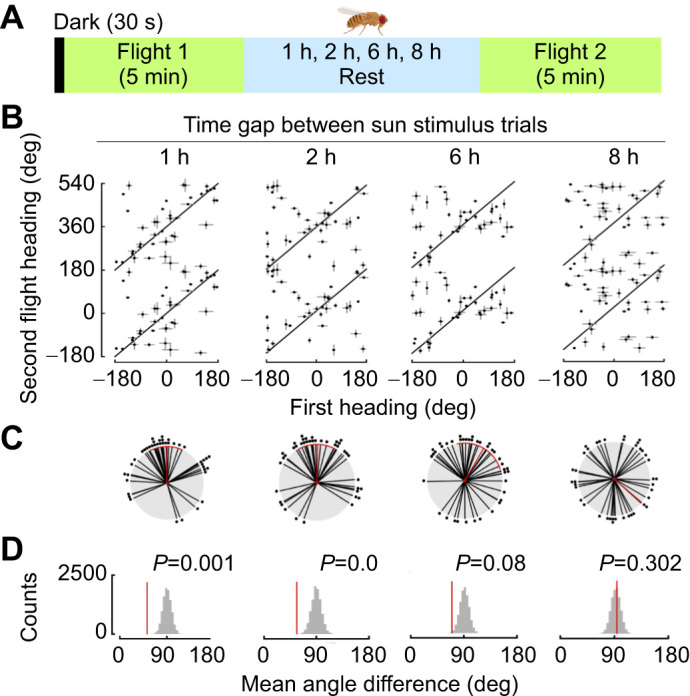
**Flies maintain heading for hours when the green LED stimulus is not moved.** (A) Experimental paradigm for time gap experiments. (B) Linear plot of heading change between Flight 1 and Flight 2 for each intertrial interval. (C) Polar plot of heading differences for each different intertrial interval (mean±95% CI, variance). (D) Reshuffled distributions of heading differences between random pairings of first and second trials. Plotting conventions as in Figs 1 and 2.

Given that flies robustly maintain their headings over short time intervals when the LED position is constant (Fig. 2), we next explored how long flies can maintain their headings in the rotating tether arena, when allowed to rest in the dark for 1, 2, 6 or 8 h between two flight bouts (Fig. 3A). Similar to findings from a rigid tether flight arena (Giraldo et al., 2018), we found that flies maintained their heading between 2 and 6 h. To assess when flies no longer maintained the same heading, we used our shuffled heading approach (Fig. 3D). We found that the heading change was smaller than expected by chance when the period of rest was 1 or 2 h (Fig. 3B–D, 1 h: mean=−1.55 deg, variance=0.51, *P*=0.001; 2 h: mean=1.12 deg, variance=0.56, *P*=0.0; 6 h: mean=30.44 deg, variance=0.67, *P*=0.08; 8 h: mean=129.86 deg, variance=0.95, *P*=0.302). However, by 6 h, flies began to change heading and by 8 h there was no relationship between the first and second heading direction, consistent with results from Giraldo et al. (2018).

### Flies do not adjust heading for a small or gradual LED position change

**Fig. 4.**
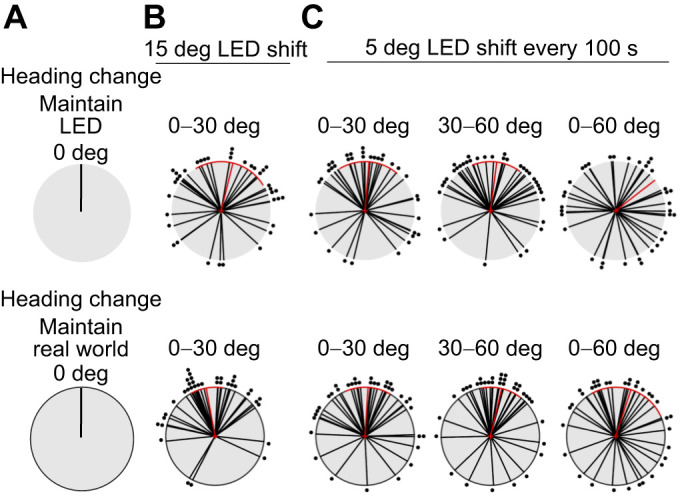
**Flies do not track small or gradual green stimulus movements.** (A) Expected outcome for heading differences when flies maintain their heading relative to the green LED (top) or arena (real world; bottom). Polar plots of heading differences with respect to a real-world reference frame are marked with a black outline. (B) Heading differences relative to the LED and absolute heading differences between minutes 0–5 and 10–15 when the sun was shifted 15 deg every 5 min. Flies showed only small differences in heading change (*n*=37, mean±95% CI, Watson's *U*^2^=0.2404, *P*=0.0174). (C) Flies showed no significant difference in relative versus absolute heading differences when the LED shifted 5 deg every 100 s, covering an angular distance of 0–60 deg (*n*=35, mean±95% CI, 0–30 deg: Watson's *U*^2^=0.03, *P*=1; 30–60 deg: Watson's *U*^2^=0.14, *P*=0.13; 0–60 deg: Watson's *U*^2^=0.08, *P*=0.4). Plotting conventions as in Fig. 1B.

Because we found that a 90 or 180 deg LED position change disrupted heading maintenance, we tested whether *Drosophila* maintain their heading relative to a green LED or relative to the arena (real-world) in response to a small or gradual stimulus change. We expected that if flies track the movement of the LED, the heading differences for the flies' heading relative to the stimulus would be centered on 0 deg (Fig. 4A). If flies instead ignored the stimulus shift and kept moving in their original direction, then we would expect the flies' heading change to be centered on 0 deg relative to the arena (Fig. 4A). First, we designed a 15 min flight trial where there were three consecutive orientation periods (Flight 1, Flight 2, Flight 3), in which the stimulus was moved 15 deg clockwise or counterclockwise between each presentation (Fig. 4B). During all orientation periods, the flies adopted arbitrary headings as in previous experiments (Flight 1: mean=−64.2 deg, variance=0.64; Flight 2: mean=−73.6 deg, variance=0.84; Flight 3: mean=−45.6 deg, variance=0.98). Because flies exhibited ∼10 deg change in heading even when we did not move the LED (data from Fig. 2C–F), we did not examine heading changes for stimulus movements of less than 30 deg. We tested whether these heading changes relative to the LED or the real world differed using a Watson's *U*^2^ test [Fig. 4B: *n*=37, heading change (LED): mean=13.8 deg, variance=0.68, Rayleigh=0.02, heading change (real-world): mean=−10.4 deg, variance=0.35, Rayleigh<0.01, Watson's *U*^2^=0.2404, *P*=0.0174]. Although the groups differed significantly, this likely reflects the smaller variance of the real-world heading changes. These data suggest that flies may be slightly more likely to maintain real-world headings, although this effect appears to be small.

Next, we asked whether flies could track the green LED when the stimulus shifted gradually. After the initial LED stimulus presentation, we shifted the illuminated LED by 5 deg every 100 s for a total angular movement of 60 deg. We compared the flies' LED-relative and real-world heading changes for each 30 deg LED shift interval (Fig. 4C; *n*=35, 0−30 deg LED: mean=4.7 deg, variance=0.61; 0–30 deg real-world: mean=3.6 deg, variance=0.51; 30–60 deg LED: mean=7.4 deg, variance=0.54; 30–60 deg real-world: mean=13.3 deg, variance=0.45) and assessed statistical differences between the LED-relative versus real-world heading changes using a Watson's *U*^2^ test (0−30 deg: Watson's *U*^2^=0.03, *P*=1; 30−60 deg: Watson's *U*^2^=0.14, *P*=0.13). We found that the mean heading change was similar between the two groups, suggesting that flies changed their heading slightly during this time period. However neither tracking the stimulus nor maintaining real-world hearings was a close fit to our observed data. Similarly, when we examined heading change over the full 60 deg, we found widely distributed headings that were not better explained by flies matching real-world or LED-relative headings (Fig. 4C; 0–60 deg LED: mean=51.9 deg, variance=0.83; 0−60 deg real-world: mean=18.1 deg, variance=0.68, Watson's *U*^2^=0.08, *P*=0.4). Although visual inspection appears to indicate that flies maintain their real-world headings during small and gradual LED movements, this was not statistically significant. In conclusion, *D. melanogaster* do not adjust their heading to track either small or gradual green LED movements.

### Flies do not possess a time-compensated sun compass to a green LED

**Fig. 5.**
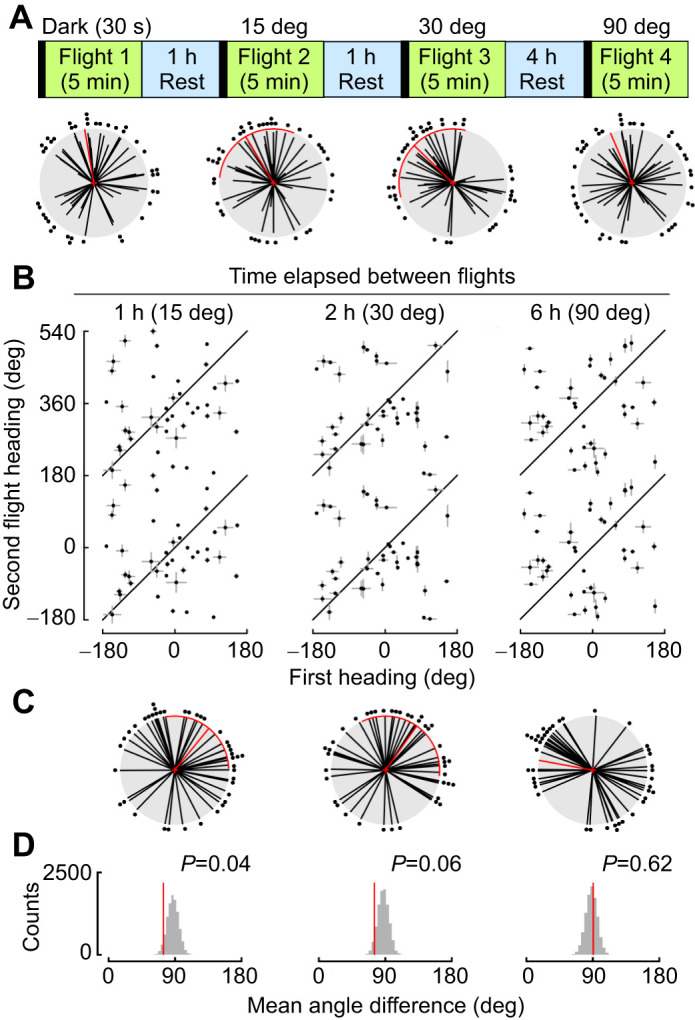
**Flies do not show evidence of a time-compensated sun compass in response to a green LED.** (A) Experimental paradigm to test whether flies use a time-compensated sun compass in response to a green LED. Headings relative to the LED position are represented in a polar plot below each flight period (*n*=41, population mean±95% CI). (B) Linear plot of heading differences between 1, 2 and 6 h. (C) Polar plots of heading change (mean±95% CI, variance). (D) Reshuffled distributions of headings between 1, 2 and 6 h trials. Plotting conventions as Figs 1 and 2.

Because flies did not track small or gradual LED position changes (Fig. 4), we asked whether flies possessed a time-compensated sun compass in response to the movement of a green LED. First, we tested a fly with a green LED for a 5 min flight. Each fly was subsequently tested 3 more times, 1, 2 and 6 h later, during which the LED position was moved at a rate of 15 deg h^−1^ to match the rate of the sun's angular movement during the day (Fig. 5A; Flight 1: mean=−9.7 deg, variance=0.84; Flight 2: mean=−30.9 deg, variance=0.73; Flight 3: mean=−45.9 deg, variance=0.75; Flight 4: mean=−23.4 deg, variance=0.86). Because movements of 15 deg are small and sun orientation behavior varies among individuals, as expected, we found that flies changed heading little after 1 h (Fig. 5B–D). However, when we examined the heading change after 2 h (30 deg) and 6 h (90 deg), we found that flies changed their headings as indicated by both our reshuffling test and a Rayleigh test for uniform distributions (Fig. 5B–D; 1 h: mean=39.3 deg, variance=0.71, Rayleigh *P*=0.03, reshuffling *P*=0.04; 2 h: mean=35.2 deg, variance=0.75, Rayleigh *P*=0.015, reshuffling *P*=0.06; 6 h: mean=−79.7 deg, variance=0.92, Rayleigh *P*=0.76, reshuffling *P*=0.62), much as when these movements occurred rapidly (Fig. 2). Collectively, this indicates that a green LED stimulus is not sufficient for sun orientation or a time-compensated sun compass when flies are able to rotate freely.

### Flies prefer to maintain orientation to a UV stimulus

**Fig. 6.**
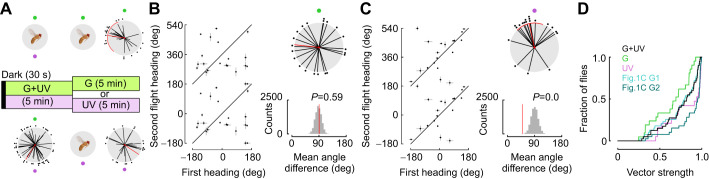
**Flies prefer to maintain orientation to a UV LED when presented with paired green and UV stimuli.** (A) Experimental paradigm to test whether flies' heading changes when presented with paired green and UV stimuli and to determine whether flies exhibit a cue preference. All headings are plotted relative to the green LED (plotted at 0 deg) and the UV LED (plotted at 180 deg; *n*=40, mean±95% CI, variance). Second period headings are separated depending on wavelength. (B) Heading changes when a green LED is presented during the second flight plotted as both linear and polar plots (mean±95%CI, variance). Reshuffled distribution of headings between first and second flight periods. (C) Heading changes when a UV LED is presented during the second flight trial. (D) Histogram of vector strength between groups of flies exposed to different visual stimuli: G±UV=paired green and UV LEDs (A), G=single green LED (A), UV=single UV LED (A), Fig. 1C G1=green LED, first 5 min (Fig. 1C), Fig. 1C G2=green LED second 5 min (Fig. 1C); mean+coefficient of variation (CV). Plotting conventions as in Figs 1 and 2.

Sunlight has multiple wavelengths that are differentially represented in the solar and anti-solar hemispheres of the sky. Thus, we tested whether flies exhibited a wavelength preference by adding a UV LED 180 deg apart from the green LED. We designed an experiment similar to a study on dung beetles (el Jundi et al., 2015a) in which both stimuli were presented for the first 5 min and one of the stimuli was turned off for the second 5 min of the trial (Fig. 6A; G+UV: mean=−147.5 deg, variance=0.82; G: mean=−145.1 deg, variance=0.84; UV: mean=30.3 deg, variance=0.81). As there was no change in LED position for either LED, a difference in heading for either stimulus would indicate a cue preference. We compared the heading change between the first and second stimulus presentations and found flies changed heading when the second LED was green (Fig. 6B; mean=−83.8 deg, variance=0.93, resampling *P*=0.59). This is a striking difference from previous findings with a stationary green LED in which flies maintained their heading (Figs 1C, 2C–J and 3B–D), suggesting that flies do not use the green LED to select a heading when both green and UV are available. In contrast, we found that flies maintained their headings when the second stimulus was UV (Fig. 6C; mean=−12.5 deg, variance=0.43, resampling *P*=0.0).

To further examine whether the change in spectral cues affected heading maintenance, we plotted a cumulative histogram of vector strengths for the duration of each flight as in Warren et al. (2018). We found that the vector strength decreased when a green LED was presented during the second flight but increased slightly when the second LED presented was UV [Fig. 6D; G+UV: mean=0.76, coefficients of variation (CV)=0.85; G: mean=0.64, CV=0.34; UV: mean=0.81, CV=0.25]. This was in contrast to earlier experiments (Fig. 1C) in which the green LED remained in the same position and vector strength increased for the second flight period (Fig. 6D; Fig. 1C G1: mean=0.74, CV=0.29; Fig. 1C G2: mean=0.87, CV=0.21). Thus, when given a choice between green and UV stimuli, flies prefer to orient and maintain their heading relative to a UV stimulus.

### Flies do not track a UV stimulus when LED position changes

**Fig. 7.**
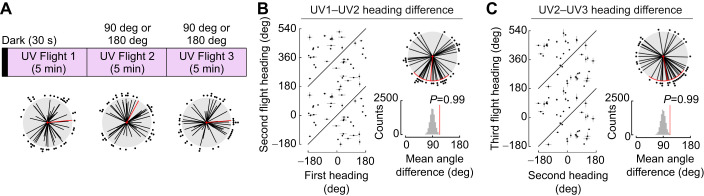
**Flies do not track large movements of a UV stimulus.** (A) Tethered flies were presented with a UV LED that was moved 90 or 180 deg after 5 min, as in Fig. 1D. Headings for each 5 min flight period were plotted relative to the UV LED, plotted at 0 deg (*n*=40, mean±95% CI). (B) Heading differences between UV Flight 1 and UV Flight 2. Reshuffled distribution of headings between UV Flight 1 and UV Flight 2 are shown below the polar plot. (C) Heading differences between UV Flight 2 and UV Flight 3 (mean±95% CI, variance). Reshuffled distribution of headings between UV Flight 2 and UV Flight 3 are shown below the polar plot. Plotting conventions as in Figs 1 and 2.

As we found that flies preferred to orient and maintain their heading relative to a UV stimulus, we next tested whether flies would maintain their heading relative to a UV LED alone when the LED position changed by 90 or 180 deg (Fig. 7A; UV Flight 1: mean=84.7 deg, variance=0.91; UV Flight 2: mean=28.5 deg, variance=0.86; UV Flight 3: mean=85.6 deg, variance=0.92). Similar to the green stimulus, flies selected a new heading when the LED position changed (Fig. 7B,C; UV1–UV2: mean=−175.2 deg, variance=0.69, reshuffling *P*=0.99; UV2–UV3: mean=169.3 deg, variance=0.7, reshuffling *P*=0.99). We compared the change in heading with a UV LED with the change in heading with a green LED (data from Fig. 1D, flights 1–3) and found that there was no significant difference between responses to the two wavelengths (Flight 1–2 heading difference: Watson's *U*^2^=0.05, *P*=0.78; Flight 2–3 heading difference: Watson's *U*^2^=0.07, *P*=0.5). Thus a single UV stimulus was not sufficient for flies to maintain heading in response to large stimulus movements.

### Flies perform sun orientation when both green and UV stimuli are available

**Fig. 8.**
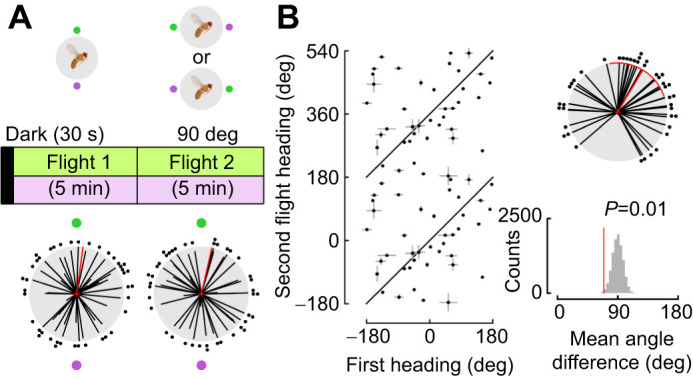
**Flies track cue movement when both green and UV wavelengths are available.** (A) Experimental paradigm to test whether flies maintain their heading relative to celestial cues when both green and UV LEDs are present. All headings are plotted relative to the green stimulus at 0 deg and the UV stimulus at 180 deg (*n*=41, mean±95% CI). (B) Heading differences between Flight 1 and Flight 2 plotted as both linear and polar plots (mean±95% CI, variance). Reshuffled distribution of headings between Flight 1 and Flight 2 are shown below the polar plot. Plotting conventions as in Figs 1 and 2.

Next, we examined whether the paired green–UV stimuli, which could mimic the spectral layout of the solar and anti-solar hemisphere of the sky, provided flies with sufficient visual information to perform sun orientation. Flies were tested for a 5 min flight trial followed by a second 5 min trial when we moved the paired stimuli 90 deg either clockwise or counterclockwise (Fig. 8A; Flight 1: mean=7.8 deg, variance=0.94; Flight 2: mean=12.9 deg, variance=0.95). In contrast to single wavelength presentations, we found that flies tracked the movement of the paired cues when both green and UV stimuli were available (Fig. 8B; heading difference: mean=30.9 deg, variance=0.67, reshuffled *P*=0.01). We concluded that the presence of paired green–UV LEDs is sufficient for flies to perform sun orientation. Indeed, in the presence of either green or UV alone, flies perform menotaxis but not sun orientation.

### Flies do not possess a time-compensated sun compass even with paired green–UV cues

**Fig. 9.**
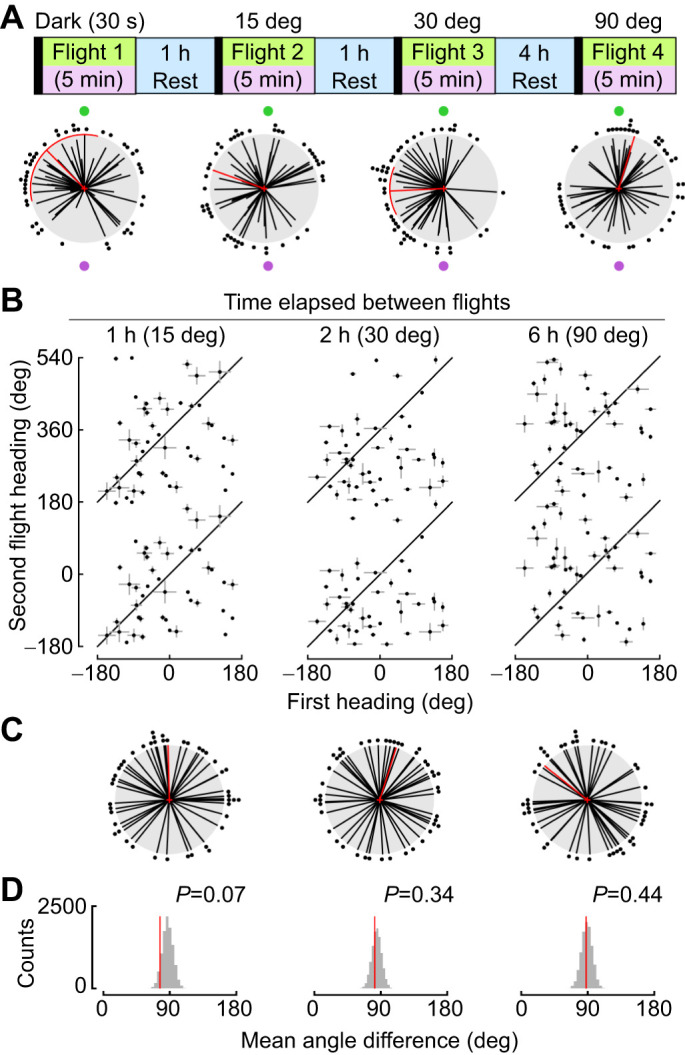
**Flies do not show evidence of a time-compensated sun compass with dual UV–green cues.** (A) Experimental paradigm to test whether flies adjust their heading for stimulus movements that occur at the pace of solar movements when presented with paired green–UV stimuli, as in Fig. 5A. Headings relative to the LED position are represented in a polar plot below each flight period, with the green LED placed at 0 deg (*n*=45, mean±95% CI). (B) Linear plot of heading differences between the first flight and subsequent trials 1, 2 and 6 h later. (C) Polar plots of heading change for data in B (mean±95% CI, variance). (D) Reshuffled distributions of heading differences for data in B and C. Plotting conventions as Figs 1 and 2.

Given that flies could maintain their heading during a 90 deg LED position change of paired green–UV stimuli (Fig. 8), we asked whether flies showed evidence of a time-compensated sun compass with these stimuli. We used an experimental paradigm similar to Fig. 5, with a UV stimulus set 180 deg from the green LED (Fig. 9A; Flight 1: mean=−44.6 deg, variance=0.76; Flight 2: mean=−70.3 deg, variance=0.82; Flight 3: mean=−92.6 deg, variance=0.54; Flight 4: mean=16.8 deg, variance=0.86). We found that similar to experiments with a single green LED (Fig. 5B,C), heading differences between each flight were uniformly distributed even after 1 h (Fig. 9B,C), compared with when the LED position remained the same (Fig. 3B,C). Differences in heading increased as time and the degree of LED position change increased (Fig. 9B–D; 1 h: mean=−1.4 deg, variance=0.82, reshuffling *P*=0.07; 2 h: mean=17.5 deg, variance=0.88, reshuffling *P*=0.34; 6 h: mean=−51.5 deg, variance=0.98, reshuffling *P*=0.44).

**Fig. 10.**
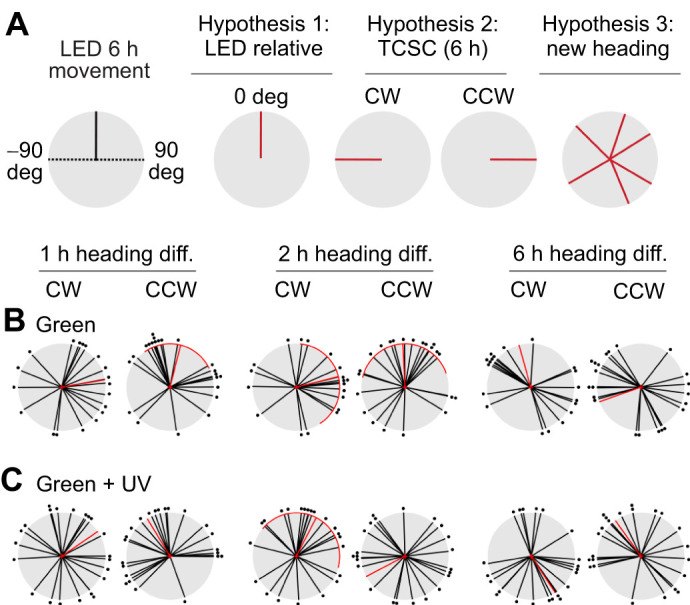
**Flies do not exhibit a time-compensated sun compass, instead selecting new headings after 6** **h.** (A) Experimental protocol in which the LED stimulus was moved a total of 90 deg over the course of 6 h in a clockwise (CW) or a counterclockwise (CCW) direction. The expected outcomes for the three hypotheses are plotted as polar plots. Hypothesis 1 indicates the predicted heading change clustered around 0 deg if flies maintain their headings relative to the stimuli. Hypothesis 2 shows that if flies possess a time-compensated sun compass (TCSC), the heading difference should be −90 deg when the LED moves clockwise and 90 deg when the LED moves counterclockwise. Hypothesis 3 shows that if flies select a new heading, heading differences should be broadly distributed. (B) Heading differences between 1, 2 and 6 h intervals separated by CW or CCW movement of the green LED (CW: *n*=19, CCW: *n*=22, mean±95% CI; data from Fig. 5). (C) Heading differences for movement of paired green–UV cues (CW: *n*=22, CCW: *n*=23, mean±95% CI; data from Fig. 9).

To test whether the lack of a time-compensated sun compass could be masked by combined clockwise and counterclockwise stimulus rotations, we set out to test three hypotheses for flies' responses to our 15 deg h^−1^ stimulus movement: maintaining an LED-relative heading, possessing a time-compensated sun compass or selecting a new heading (Fig. 10A). We separated our 15 deg h^−1^ data for both green and paired green–UV stimuli into clockwise and counterclockwise rotations. If flies maintained their headings relative to the stimuli, we expected that heading changes would be clustered around 0 deg. Alternatively, if flies possessed a time-compensated sun compass, heading differences at 6 h would be clustered around −90 and 90 deg for the clockwise and counterclockwise groups, respectively. Finally, if flies selected new headings, we expected broad heading difference distributions. For both the green and paired green–UV cues, heading changes did not cluster around 0 deg or 90/−90 deg (Fig. 10B; 6 h CW mean=−15.3 deg, variance=0.91; CCW mean=−109.4 deg, variance=0.86; Fig. 10C; 6 h CW mean=146.8 deg, variance=0.78; CCW mean=−35.4 deg, variance=0.76). Furthermore, there were no significant differences between clockwise and counterclockwise groups for either green or dual cues (green 6 h CW–CCW: Watson's *U*^2^=0.06, *P*=0.6; green–UV 6 h CW–CCW: Watson's *U*^2^<0.001, *P*=1). The mean heading differences for 1 and 2 h intervals also did not align with similar predictions for a time-compensated sun compass, although the differences would be harder to detect (Fig. 10B; 1 h CW: mean=80.2 deg, variance=0.71; 1 h CCW: mean=14.3 deg, variance=0.62; 2 h CW: mean=75.8 deg, variance=0.66; 2 h CCW: mean=−2.7 deg, variance=0.69; Fig. 10C; 1 h CW: mean=55.5 deg, variance=0.82; 1 h CCW: mean=−31.2 deg, variance=0.71; 2 h CW: mean=27.3 deg, variance=0.7; 2 h CCW: mean=−117.7 deg, variance=0.93). Furthermore, given that flies do not respond to the green LED as the sun but perform sun orientation with paired green–UV cues, we expected we would find differences in heading changes between groups. Instead, we found no significant difference between green alone and green–UV (1 h: Watson's *U*^2^=0.05, *P*=0.7; 2 h: Watson's *U*^2^=0.04, *P*=0.9; 6 h: Watson's *U*^2^=0.09, *P*=0.37). Collectively, these analyses indicate that in response to 15 deg h^−1^ movement of either paired or single LED stimuli, flies neither maintain their real-world headings nor show heading characteristic of a time-compensated sun compass; instead, flies select new headings.

## DISCUSSION

Here, we provide the first demonstration that *Drosophila melanogaster* robustly perform sun orientation in response to a stimulus with multiple wavelength cues in a flight arena in which the fly can rotate about its vertical axis. This experimental design provides a more naturalistic flight experience for the fly in which it can experience body rotations. Indeed, the presence of body rotations can have a dramatic impact on visually guided behavior, as demonstrated for responses to a vertical bar in *Drosophila* (Rimniceanu et al., 2023). While sun orientation in response to a green spot has been explored in a rigid tether (Giraldo et al., 2018), in that setup, the experimenter lacks the ability to control the stimulus position and rate of movement during orientation flights. Thus, the rotating tether allowed us to test the magnitude and speed of stimulus movement and explicitly test for a time-compensated sun compass. Combined with the presentation of both green and UV wavelengths, we uncovered previously undescribed aspects of *Drosophila* sun orientation.

We found that in the presence of rotational cues, flies required a specific combination of wavelengths – green and UV separated by 180 deg – to track changes in LED position. In response to a green LED, flies maintained the same heading under a variety of conditions – continuous flight, stopping flight for hours, and experiencing darkness – provided that the LED stayed in the same position. In addition, flies did not maintain the same heading with respect to a green LED in response to small or gradual changes in stimulus position. Indeed, even when we moved the illuminated green LED at the rate of natural sun movement, 15 deg h^−1^, flies did not maintain the same relative heading. However, in response to large movements (>90 deg) of a green LED, flies tended to maintain their real-world heading, at least over short intervals. Because flies do not respond to a green LED as a sun and thus are not performing sun orientation, the interpretation of this finding is not immediately clear. We can speculate that in response to large-scale movements of an object used for menotaxis, flies switch to rely on proprioceptive cues. However, the importance of a change in cue prioritization for flight under natural conditions awaits further study. Together, our findings suggest that a green spot is not sufficient to induce true sun orientation in flies, despite its efficacy for other taxa (el Jundi et al., 2015a,b; Beetz et al., 2022). Experimentally, this suggests that researchers should use caution in interpreting how a given stimulus is perceived by the animal, particularly as requirements for sun orientation are likely taxa specific (el Jundi et al., 2015a,b).

As the sun creates celestial cues that are more complex than a small, bright green spot, we added a UV light stimulus 180 deg apart from the green LED to simulate the antisolar hemisphere, allowing for a more accurate representation of the chromatic variation visible to flies in the sky. Although flies preferred to select and maintain their orientation to the UV light stimulus when first presented with green–UV paired stimuli, the UV stimulus alone was not sufficient for flies to maintain their original heading during a stimulus position change. Instead, flies require both green and UV cues to maintain the same heading relative to visual cues in response to a large, rapid LED position change, a canonical test of celestial compass orientation across insects (e.g. Weir and Dickinson, 2012; el Jundi et al., 2014). Given that flies do perform sun compass orientation with paired UV–green cues, we set out to test whether they exhibited a time-compensated sun compass. We found that even with this additional chromatic information, flies did not maintain the same heading relative to our LEDs over the course of hours when the LED position was moved 15 deg h^−1^. Indeed, even when separated by clockwise and counterclockwise movements, we found no evidence for a time-compensated sun compass. While a time-compensated sun compass has been demonstrated in monarch butterflies and honeybees (Merlin et al., 2009; Shlizerman et al., 2016; von Frisch and Lindauer, 1956), in each of these well-documented cases, the insects use the sun to provide real-world directional cues. In contrast, we do not have any evidence that *Drosophila* extract compass information from the position of the sun. Rather, the directional cues are likely used to maintain a straight path until flies encounter a salient odor, at which point they can orient using olfactory cues (Dickinson, 2014; Leitch et al., 2021). Although the lack of a time-compensated sun compass would mean that flies would not be able to fly perfectly straight using sun position alone, over the short distances flies likely cover in the field, this effect would be minimal. Nevertheless, it is possible that the lack of a time-compensated sun compass that we observed could be related to the absence of natural celestial cues in our laboratory-reared flies, as experience with the natural sun movement is required for a time-compensated sun compass in honeybees (Lindauer, 1960). While the role of natural skylight experience awaits future investigation, the lack of a time-compensated sun compass is consistent with our understanding of *Drosophila* ecology – non-migratory animals without a home location, similar to a suggested lack of time compensation in dung beetles (el Jundi et al., 2019). Although the origin of heading preference remains unknown in *Drosophila* or other taxa that perform menotaxis, our data provide behavioral evidence that *Drosophila* are sensitive to the chromatic content of the celestial stimulus to interpret the stimulus as the sun. These psychophysical experiments provide the basis to explore the neural mechanisms underlying orientation decisions.

The underlying neural basis for the wavelength sensitivity that we observed has yet to be identified. Although the neural circuitry underlying orientation and navigation in insects has advanced rapidly in recent years (Beetz et al., 2023; Buehlmann et al., 2020; Honkanen et al., 2019; Stone et al., 2017; Webb and Wystrach, 2016), and is especially well described in *Drosophila* (Green et al., 2017; Green et al., 2019; Kim et al., 2019; Turner-Evans et al., 2017), we are only beginning to explore the interaction among cues (Buehlmann et al., 2020). Neurons in the central complex receive chromatic light information from the anterior optic tubercle (AOTU) (el Jundi et al., 2011), and polarization-sensitive neurons in monarch butterflies are known to respond to the azimuth rather than the wavelength of light spots (Heinze and Reppert, 2011). In locusts, central complex neurons have been found to have similar tuning to green and UV stimuli (Pegel et al., 2018). Intriguingly, *Drosophila* display a strong preference for UV light during phototaxis (Fischbach, 1979; Gao et al., 2008; Hu and Stark, 1977; Karuppudurai et al., 2014), and this preference requires a class of medullar neurons called distal medulla 8 (Gao et al., 2008; Karuppudurai et al., 2014) which receive signals from UV-sensitive R7 photoreceptors (Montell et al., 1987). While this suggests a possible neural pathway that allows for flies' UV preference during paired green–UV cue experiments, it does not provide a mechanism for flies' requirement for paired green–UV wavelengths for sun orientation. Indeed, one large gap in our understanding of orientation and navigation in *Drosophila* stems from our shockingly limited knowledge of the natural history of this genetic model (Markow, 2015; Soto-Yéber et al., 2018). To effectively leverage the advances in *Drosophila* neuroscience to explain behavior, we need to ground our explanations for laboratory experiments in this species' natural history. Although their small size still presents limitations for understanding individual insect movement in the field (Kumari and Hasan, 2020), coupling laboratory experiments with field studies holds promise for advancing our understanding of *Drosophila* orientation. The remarkable conservation of neural circuitry among insect taxa has meant that advances in one species add depth to our understanding of orientation and navigation across insect taxa.

## Supplementary Material

10.1242/jexbio.246817_sup1 Supplementary information
