# Supplementary material for: Drosophila require both green and UV wavelengths for sun orientation but lack a time-compensated sun compass
Source: J Exp Biol. 2024 Oct 14;227(19):jeb246817. doi: 10.1242/jeb.246817 (PMC11529886; doi:10.1242/jeb.246817)
Supplement: Supplementary information [file jexbio-227-246817-s1.pdf]

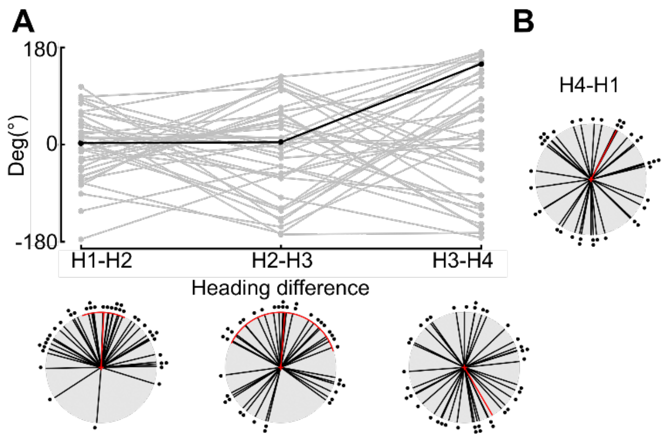

Fig. S1. Flies maintain real world headings over short time intervals when LED position changes.

(A) Real world heading differences calculated for each 5-minute flight period. Linear plot depicts heading differences over the course of flight. Grey lines indicate individual flies and black indicates the population mean. Corresponding polar plots are shown below (n=38, mean±CI, variance). (B) Heading difference between the first and last 5 minutes of the 20-minute flight period (mean±CI, variance).

Journal of Experimental Biology • Supplementary information

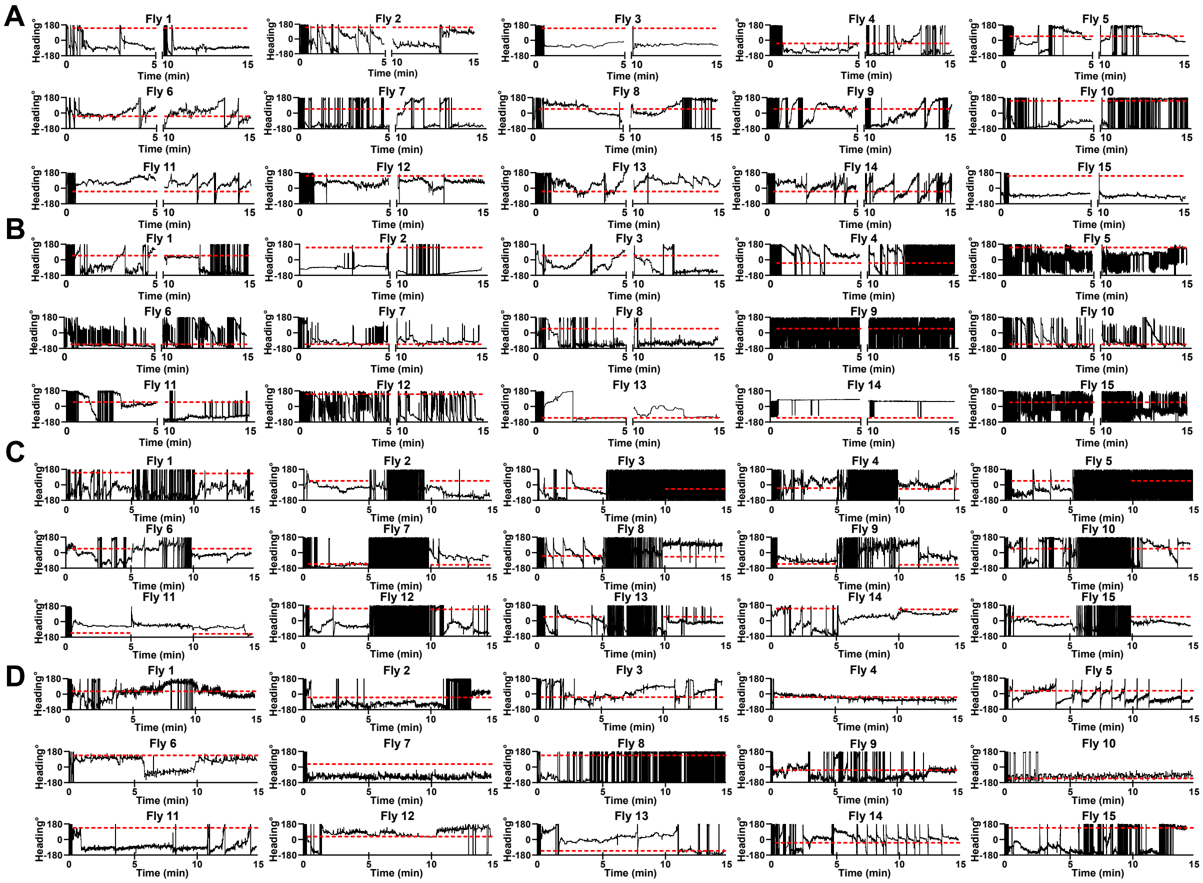

Fig. S2. Individual headings of flies when the LED position was held constant during the varied stimuli flight trials.

(A) Time series of the first 15 individual flies of Figure 2C that experienced rest in the dark during the intertrial interval. Time is indicated in the x-axis and the heading angle is shown on the y- axis in degrees. The LED position is depicted with a dashed red line. (B) Time series of the first 15 individual flies of Figure 2D, which experienced rest with an illuminated LED. (C) Time series of the first 15 individual flies that flew in the dark during the intertrial interval. From Figure 2E. (D) Time series of the first 15 individual flies of Figure 2F that flew with an illuminated LED during the intertrial interval.

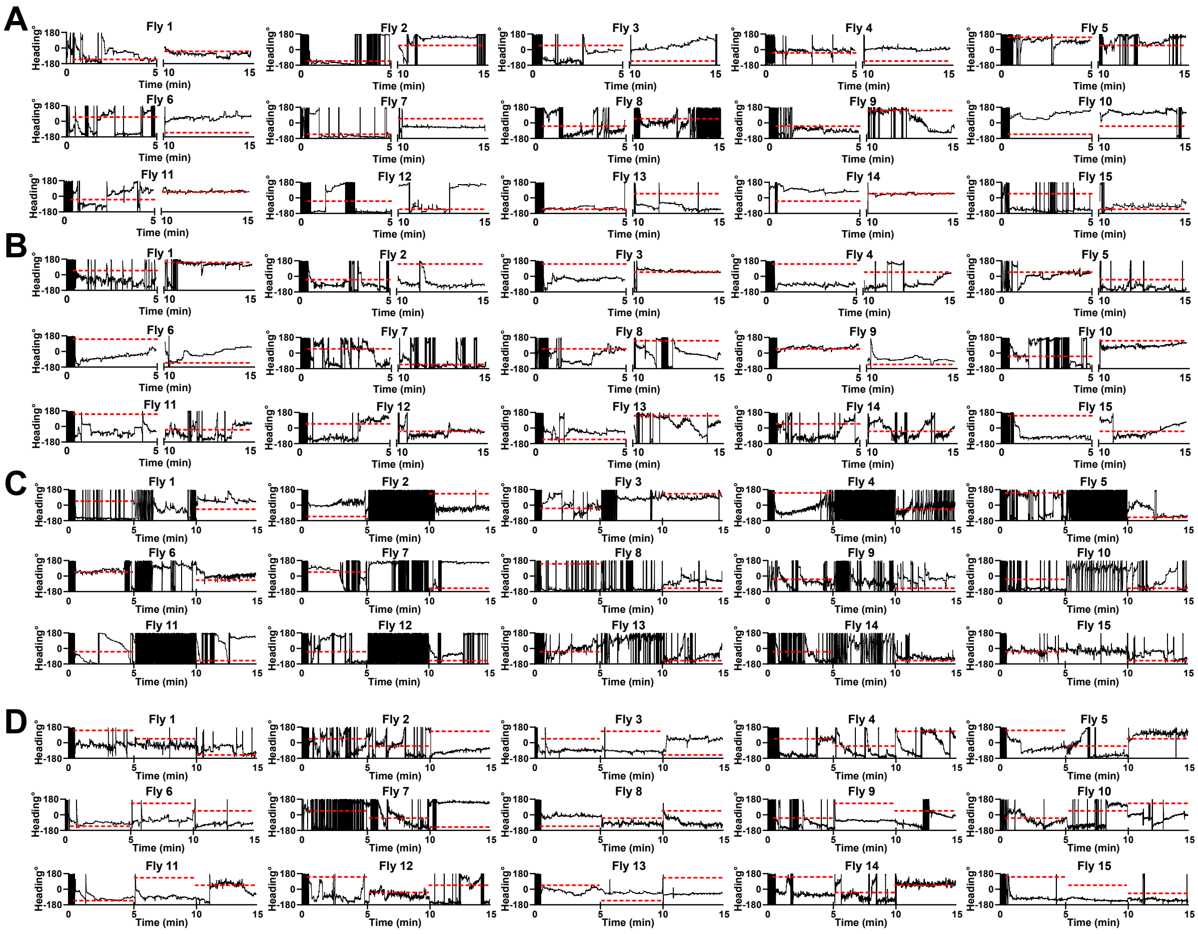

Fig. S3. Individual headings of flies when the LED position was moved 90° or 180° during the varied stimuli flight trials.

(A) Time series of the first 15 individual flies of Fig. 2K, resting in the dark during the intertrial interval. Plotting conventions are as in Fig. S2. (B) Time series of the first 15 individual flies that rested with an illuminated LED between orientation flights. From Fig. 2L. (C) Time series of the first 15 individual flies of Figure 2M that flew in the dark during the intertrial interval. (D) Time series of the first 15 individual flies that flew with an illuminated LED. From Fig. 2N.

Journal of Experimental Biology • Supplementary information

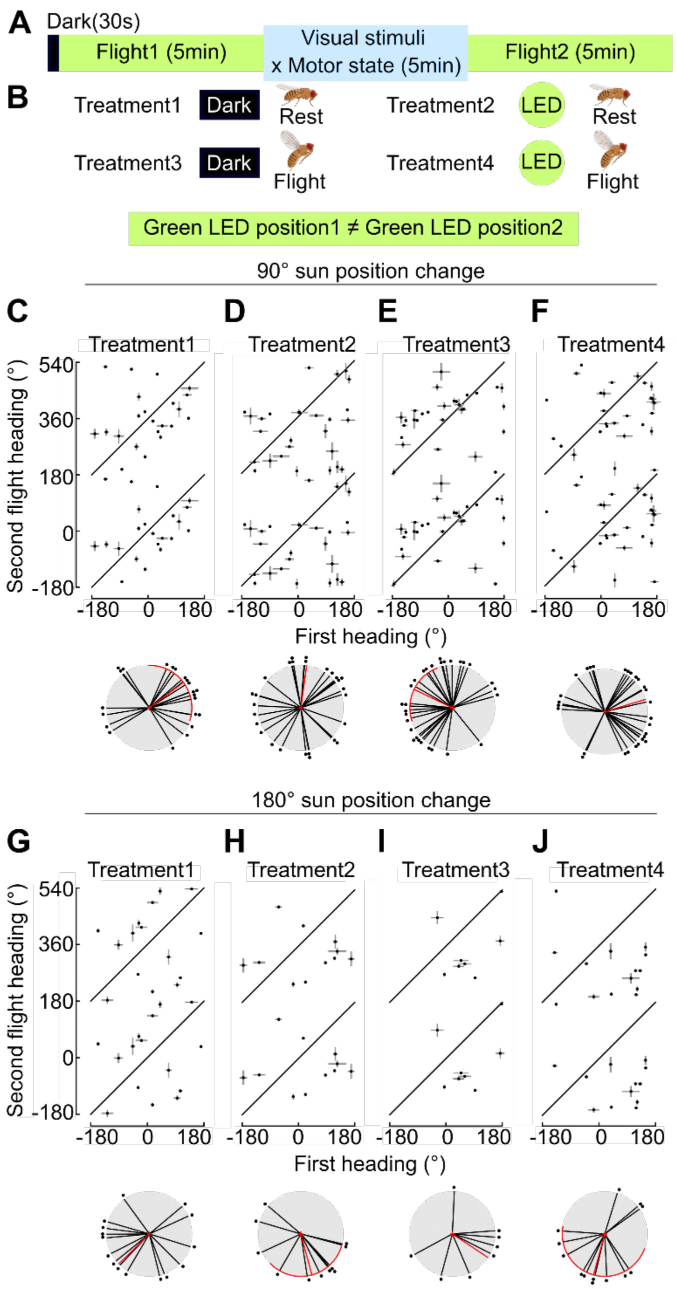

Fig. S4. Flies tend to keep real-world headings in response to 180° movement of a green LED. (A) Experimental paradigm for the varied stimuli flight trial where flies experience a brief 30s dark period followed by a 5min flight period (Flight1), an intertrial period with different combinations of motor and visual stimuli (5min), and a second 5min flight period (Flight2). (B) Four different treatment groups were used for the intertrial period which consisted of a combination of two different visual states and motor states. (C-F) Heading difference when sun position changed by 90°. Linear plots of the first flight heading (x-axis) plotted against the second flight heading (y-axis). Plotting conventions as in Figure 2C. Heading differences between the first sun presentation and the second sun presentation are plotted as polar plots below the linear plots, using the same plotting conventions as Fig. 1B (mean±CI, variance). (G-J) Heading difference when the LED position changed by 180°. Plotting conventions are as above.

Journal of Experimental Biology • Supplementary information
